# EIAV-Based Retinal Gene Therapy in the *shaker1* Mouse Model for Usher Syndrome Type 1B: Development of UshStat

**DOI:** 10.1371/journal.pone.0094272

**Published:** 2014-04-04

**Authors:** Marisa Zallocchi, Katie Binley, Yatish Lad, Scott Ellis, Peter Widdowson, Sharifah Iqball, Vicky Scripps, Michelle Kelleher, Julie Loader, James Miskin, You-Wei Peng, Wei-Min Wang, Linda Cheung, Duane Delimont, Kyriacos A. Mitrophanous, Dominic Cosgrove

**Affiliations:** 1 Boys Town National Research Hospital, Omaha, Nebraska, United States of America; 2 Oxford BioMedica (UK) Ltd, Oxford Science Park, Oxford, United Kingdom; 3 University of Nebraska Medical Center, Omaha, Nebraska, United States of America; Institut de la Vision, France

## Abstract

Usher syndrome type 1B is a combined deaf-blindness condition caused by mutations in the *MYO7A* gene. Loss of functional myosin VIIa in the retinal pigment epithelia (RPE) and/or photoreceptors leads to blindness. We evaluated the impact of subretinally delivered UshStat, a recombinant EIAV-based lentiviral vector expressing human *MYO7A*, on photoreceptor function in the *shaker1* mouse model for Usher type 1B that lacks a functional *Myo7A* gene. Subretinal injections of EIAV-CMV-GFP, EIAV-RK-GFP (photoreceptor specific), EIAV-CMV-MYO7A (UshStat) or EIAV-CMV-Null (control) vectors were performed in *shaker1* mice. GFP and myosin VIIa expression was evaluated histologically. Photoreceptor function in EIAV-CMV-MYO7A treated eyes was determined by evaluating α-transducin translocation in photoreceptors in response to low light intensity levels, and protection from light induced photoreceptor degeneration was measured. The safety and tolerability of subretinally delivered UshStat was evaluated in macaques. Expression of GFP and myosin VIIa was confirmed in the RPE and photoreceptors in *shaker1* mice following subretinal delivery of the EIAV-CMV-GFP/MYO7A vectors. The EIAV-CMV-MYO7A vector protected the *shaker1* mouse photoreceptors from acute and chronic intensity light damage, indicated by a significant reduction in photoreceptor cell loss, and restoration of the α-transducin translocation threshold in the photoreceptors. Safety studies in the macaques demonstrated that subretinal delivery of UshStat is safe and well-tolerated. Subretinal delivery of EIAV-CMV-MYO7A (UshStat) rescues photoreceptor phenotypes in the *shaker1* mouse. In addition, subretinally delivered UshStat is safe and well-tolerated in macaque safety studies These data support the clinical development of UshStat to treat Usher type 1B syndrome.

## Introduction

Usher syndrome (USH) is a genetically heterogeneous disorder characterized by congenital deafness associated with delayed onset and progressive retinitis pigmentosa (RP). Ten genes identified as causative for the disorder are grouped into three clinical subtypes based on the degree of hearing loss and the presence or absence of vestibular areflexia [1]–[12]. Usher syndrome type I, the most severe clinical sub-type, is characterized by total congenital deafness associated with early onset and rapidly progressing RP. Of the six genes linked to clinical sub-type I, the gene associated with USH1B is the most commonly mutated. This gene encodes a non-conventional myosin motor protein, myosin VIIa.

Several mouse models containing spontaneous or induced mutations in the USH1B gene have been described [13]. All of these mice are congenitally deaf, but none show signs of retinal degeneration, raising questions as to how appropriate these mice are for studying retinal dysfunction in USH1B [14], [15]. A small (approximately 20%) decrease in electroretinography (ERG) amplitudes has been demonstrated however b-wave thresholds do not vary significantly from strain/age-matched wild type mice [16].

In normal photoreceptors, the G protein α-transducin translocates from the outer to the inner segments in response to light and then back to the outer segments upon dark adaptation [17]. This process is thought to provide a neuroprotective buffering mechanism for the photoresponse, allowing greater light sensitivity under low light conditions, and reduced sensitivity under bright light conditions [18], [19]. The light threshold needed for the translocation of α-transducin is elevated under certain pathological conditions and may be associated with a sensitivity to light induced photoreceptor cell degeneration [20]–[23]. For example, we recently reported that the *shaker1* mouse model for USH1B displays significantly elevated thresholds for light induced α-transducin translocation from the outer segments to the inner segments. Furthermore, when exposed to an acute, continuous six day moderate intensity light (2500 lux), or to a chronic, moderate intensity light/dark cycle (1500 lux), *shaker1* mice display a robust retinal photoreceptor degenerative phenotype compared to age/strain-matched wild type mice [21].

These retinal readouts provide useful assays for functional rescue of the *shaker1* model and to further the understanding of retinal pathology in Usher syndrome type 1B. In this study, we evaluate the potential of an EIAV-based lentiviral gene therapy vector, EIAV-CMV-MYO7A (UshStat), which expresses the functional human myosin VIIa protein to rescue these phenotypes in the *shaker1* mouse model of USH1B. Following subretinal delivery of the UshStat vector, myosin VIIa expression was detected in the retinal pigment epithelium (RPE) and photoreceptor cells, both of which normally express myosin VIIa and both of which may be required to maintain photoreceptor health in individuals with USH1B. There was also restoration of the normal light threshold for α-transducin translocation in the *shaker1* mice and the photoreceptors were protected from acute (2000 lux continuous/6 day) and chronic (1500 lux light/dark cycle/3 months) light induced cell degeneration.

The toxicity, biodistribution and shedding characteristics of the UshStat vector were examined over three months following a single subretinal injection in macaques and the data show that the vector is safe and well-tolerated, and localized to the site of administration in the eye.

These studies suggest UshStat is capable of functional rescue and may prove to be an effective therapy for preventing light dependent retinal degeneration in humans with Usher syndrome type IB.

## Materials and Methods

### Animals

*shaker1* mice (*Myo7a^sh1-11J^*) were purchased from Jackson Laboratories (Bar Harbor, ME; http://jaxmice.jax.org/strain/005468.html). These mice were back-crossed 9 generations onto the 129 Sv/J background. Wild type 129 Sv/J mice were used as controls for all studies. The RPE65 transcript for this strain was sequenced and found to be of the L450 genotype and thus positive for a quantitative trait *locus* that confers light sensitivity to photoreceptors [24]. The animals were kept at the Boys Town National Research Hospital *vivarium* in transparent cages under 12 hour light/dark cycle. Procedures for handling animals followed NIH guidelines and were in accordance to an approved institutional IACUC protocol. Every effort was made to minimize discomfort and distress.

The safety study was performed on naïve adult male and female (2–4 years old) Rhesus macaques (*Macaca mulatta*). All animal studies were in accordance with the United States Food and Drug Administration (FDA) GLP (Good Laboratory Practice) regulations. The NHP (Non-Human Primate) GLP tox/biodistribution study was approved by the IACUC committee (or equivalent) at Charles River Laboratories, (Charles River Preclinical Services, Senneville, Montreal, Canada). The macaques were socially housed (up to 3 animals of same sex and same dosing group together) in stainless steel cages equipped with a stainless steel mesh floor and an automatic watering valve. Animals were separated during designated procedures/activities. Animals were maintained at 21°C to 27°C temperatures, 30% to 70% humidity, and 12/12 light dark cycle. All macaques had access to a standard certified pelleted commercial primate food (2050C Certified Global 20% Protein Primate Diet: Harlan) twice daily. Municipal tap water that had been softened, purified by reverse osmosis and exposed to ultraviolet light was freely available. For psychological/environmental enrichment, macaques were provided with items such as perches, floor toys, foraging devices and/or hanging devices. Additional enrichment, such as music, natural sounds and color videos films were also provided. Each macaque was also offered food supplements (such as certified treats, fresh fruit).Veterinary care was available throughout the course of the study and macaques were examined by the veterinary staff as warranted by clinical signs or other changes. There were no unscheduled deaths during the course of the study.

### Subretinal Injection of Macaques

Topical ophthalmic antibiotic (gentamicin) was applied to both eyes twice on the day before treatment, immediately following the injection and twice on the day following the injection (A.M. and P.M.). Animals were fasted overnight prior to the dosing procedure. Prior to dosing, mydriatic drops (1% Mydriacyl and/or 2.5% phenylepherine) were applied to both eyes, as necessary. Prior to dosing, the animals received an intramuscular injection of a sedative cocktail of ketamine (15 mg/kg), glycopyrrolate (0.01 mg/kg) and xylazine (0.6 mg/kg). The animals were intubated with an endotracheal tube and anesthesia maintained with an isoflurane/oxygen mix. For both eyes, the conjunctivae were flushed with benzalkonium chloride diluted in Sterile Water, USP to 1∶10,000 (v/v). Following injection, a brief post-operative exam was performed to localize the bleb and size area and any other pertinent information. An analgesic, (buprenorphine, 0.01 mg/kg) was administered by intramuscular injection to each animal prior to the procedure or following completion of the procedure, and once again approximately 8 to 12 hours following the first administration. Additional doses at a similar interval may be administered if considered to be necessary every 8 to 12 hours following the last administration.

### Post Dose Reactive Therapy

The animals were observed daily by a trained veterinarian and palliative treatment was given for inflammation related to the dosing procedures and to provide appropriate palliation of adverse events (e.g., discomfort). This was done on an individual animal basis. Should it be necessary, 1% atropine, ofloxacin and/or nonsteroidal anti-inflammatory drug (NSAID) were administered. Ophthalmic examinations were carried out frequently on each animal to assess the impact of the subretinal dosing of the vector: once pre-treatment and on days 3, 8, 15, 22, 29 and at weeks 9 and 13 of treatment. Animals were subjected to funduscopic (direct and indirect ophthalmoscopy) and biomicroscopic (slit lamp) examinations following mydriatic administration. The mydriatic used was 1% tropicamide. A sedative, ketamine HCl for injection, U.S.P., (15 mg/kg) was administered by intramuscular injection following an appropriate fasting period. On occasions when ocular images were captured animals received an intramuscular injection of a sedative cocktail of ketamine (15 mg/kg), xylazine (0.6 mg/kg) and glycopyrrolate (0.01 mg/kg). Examinations were performed by a board-certified veterinary ophthalmologist.

### Terminal Procedure for scheduled deaths

The deaths were scheduled according to regulatory requirements for a long-term GLP toxicology study in NHPs to support the initiation of a clinical trial in man. The animals underwent exsanguination by incision of the axillary or femoral arteries following anesthesia by intravenous injection of sodium pentobarbital. A sedative, ketamine HCl for injection, U.S.P. was administered by intramuscular injection before animals were transported from the animal room to the necropsy area.

### Vector production by transient transfection

**Figure 1:**
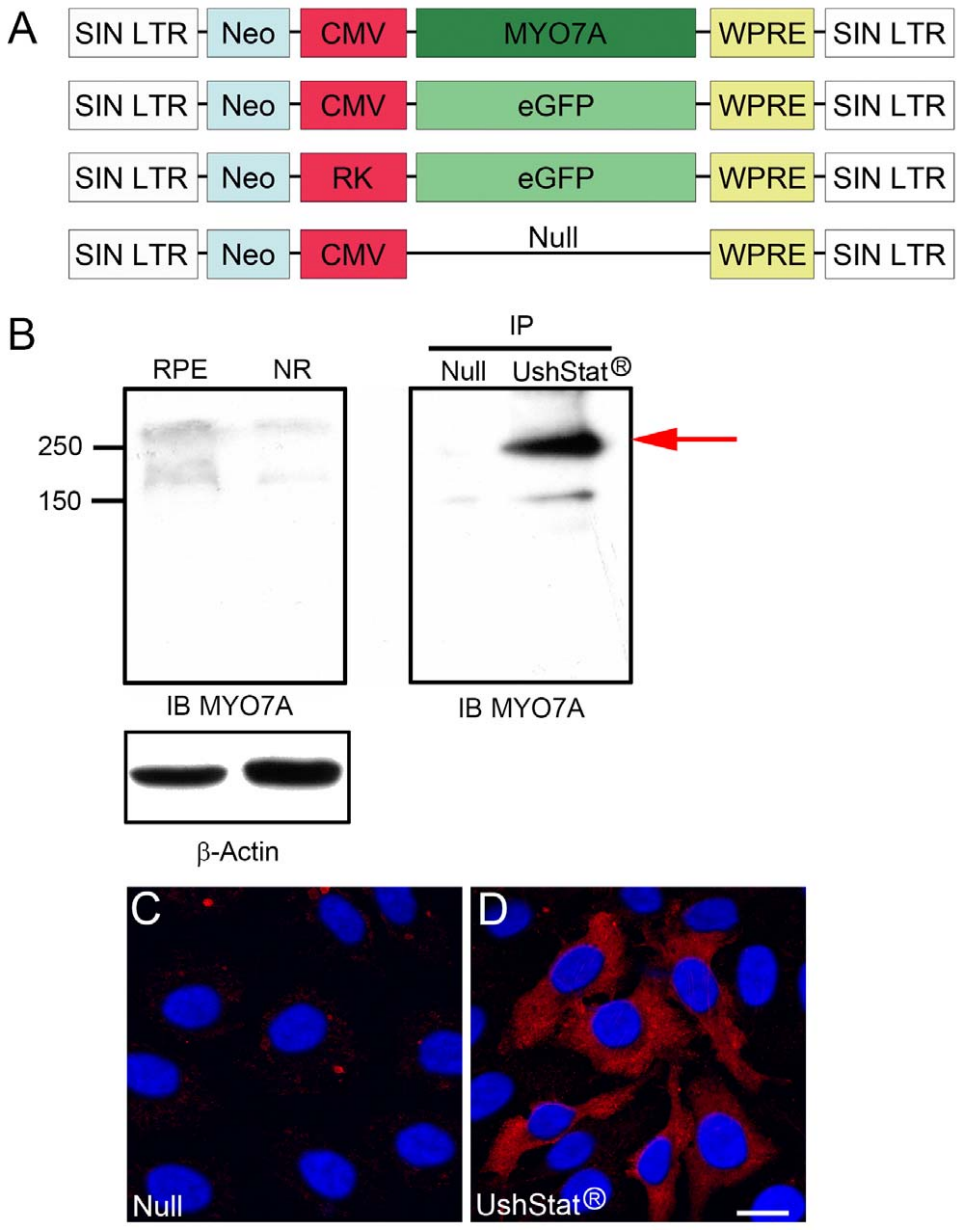
**A**. *Schematic diagram showing the genetic structure of the integrated EIAV vectors used in this study*. EIAV-CMV-MYO7A (UshStat) is based on a non-replicating non-human recombinant lentiviral vector based on the non-pathogenic wild type equine infectious anaemia virus (EIAV). The wild-type EIAV virus has 6 distinct genetic units, however, the majority of these EIAV sequences have been removed to produce a minimal vector system that contains less than 10% of the original viral genome and does not contain any viral promoters or enhancers and there are no coding regions for accessory proteins in either the EIAV genome or in the packaging system. SIN LTR: Self inactivating long term repeat. Neo: Neomycin open reading frame (ORF). CMV: Cytomegalovirus promoter (constitutive). RK: Rhodopsin kinase promoter (photoreceptor specific). eGFP: enhanced green fluorescent protein ORF. MYO7A: Myosin VIIa ORF. WPRE: Woodchuk hepatitis virus post-transcriptional regulatory element. **B**. Expression analysis of myosin VIIa in 4 weeks mouse eye and HeLa cells transfected with the EIAV-CMV-Null (Null) or UshStat constructs. β-actin was used as loading control. RPE: retinal pigment epithelium. NR: neuroretina. IP/Null: immunoprecipitates of HeLa cells transfected with the null vector. IP/UshStat: immunoprecipitates of HeLa cells transfected with the myosin VIIa vector. IB MYO7A: immunoblot with the mouse anti-myosin VIIa. Molecular weight markers are denoted to the left. **C–D**: Immunocytochemistry studies of HeLa cells transduced with the null (**C**) or the UshStat vector (**D**) and immunostained for myosin VIIa (red). DAPI was used to counter stain the nucleus. Scale bar: 15 μm.

The EIAV lentiviral vector system used to produce the EIAV vectors involves the transient transfection of human embryonic kidney 293T cells (A master cell bank (MCB) has been produced by Cobra Biomanufacturing plc, Keele, U.K. with cells derived from the Stanford University HEK293T cell stock deposited with the ATCC (SD-3515; Lot# 2634366)) with three plasmid constructs, namely the vector genome coding for *MYO7A* or *GFP* gene, the codon optimized *gag*-*pol* packaging component and the VSVG envelope packaging component, using Lipofectamine 2000 CD (Invitrogen, NY) according to the manufacturer's instructions (**Figure 1A**). Cell culture supernatants were harvested for each of the vectors and concentrated 2000-fold by centrifugation. This comprised an initial low-speed centrifugation at 6000*xg* at 4°C for a minimum of 18 hr, followed by ultracentrifugation at 50,000*xg* at 4°C for 90 minutes. The titers of the EIAV vectors used in this study were determined by integration (DNA) titer assay using the method as previously described [25]. Stock titers of the vectors were as follows: UshStat 2.1×10^7^ TU (transforming units)/mL, EIAV-CMV-Null 6.2×10^7^ TU/mL, EIAV-CMV-GFP 9.4×10^6^ TU/mL, EIAV-RK-GFP 6.0×10^8^ TU/mL.

For the macaque GLP safety study, the UshStat vector was produced in a manner analogous to the GMP (Good Manufacturing Practice) grade clinical vector in which the virus-containing supernatant was harvested and the virus was purified and concentrated by anion exchange chromatography and hollow fiber technology. This method has been described previously [26].

### Subretinal administration of EIAV vectors

The handling of animals was the same for each treatment group. Because the *shaker1* phenotype (circling behavior) is evident only in animals that are older than 3 weeks of age, all newborn mice were injected with the corresponding vector and at 3 weeks of age those that did not show circling behavior were sacrificed in accordance to an approved institutional IACUC protocol. Control animals (TSSM (tromethamine, NaCl, sucrose and mannitol) and Null injected) were treated under same conditions. Animals that demonstrated a poor health state were removed from the experimental protocol. Strategy design and end points were defined in advance to data collection and the assessing and quantification of the experimental outcomes were done using unblinded procedures.

The configuration of the EIAV-based lentiviral vectors used in this study is outlined in **Figure 1**. Note that GFP expression from either the EIAV-CMV-GFP or EIAV-RK-GFP vectors was used to delineate the region of EIAV vector transduction following the creation of a subretinal bleb. The EIAV-CMV-GFP vector contains the CMV promoter which leads to GFP expression in both photoreceptors and RPE cells, while expression from the EIAV-RK-GFP vector is restricted to the photoreceptors due to the use of the rhodopsin kinase (RK) promoter. The UshStat vector contains the CMV promoter to express the full length human myosin VIIa protein, which will be expressed in both the target photoreceptors and RPE cells. An empty vector control (EIAV-CMV-Null) is used to control for any effects of lentiviral vector transduction on experimental parameters. Control (TSSM) formulation buffer was used to control for the effects of subretinal injection.

For the subretinal administration in mice the UshStat and GFP vectors were co-injected at a final volume of 1 μl per retina. Total co-transformation units injected per μl were as follow: UshStat: 1.7×10^6^+ EIAV-CMV-GFP: 1.9×10^5^. EIAV-CMV-Null: 5×10^6^+ EIAV-RK-GFP: 1.2×10^7^. To avoid repetitive freeze/thaw cycles that may have led to a decrease in the titer, both type of viral suspensions i.e. UshStat and EIAV-CMV-GFP or EIAV-CMV-Null and EIAV-RK-GFP, were thawed once on the day of the experiment, mixed accordingly, used and the rest of the mixture aliquoted into 10 μl aliquots that were used for subsequent experiments. The targeted region for EIAV injection was inferolateral to the optic nerve. All mice obtained from the *shaker1*/wild-type breeding pairs were subretinally treated as neonates (P1-P3). However, the homozygous *shaker1* progeny can only be differentiated from heterozygotes at P15 based on the characteristic ‘head-bobbing’ phenotype of the *shaker1* mice. Only subretinally treated *shaker1* homozygous mice were processed for subsequent analyses that were performed at least 4 to 6 weeks following subretinal injection. A sub-retinal injection was considered successful when we were able to see GFP expression in the injected area of the retina by immunohistochemistry analysis. For all the experiments the area of the retinas analyzed, were within 0.2 mm from the injection site.

### HeLa cell cultures and transduction protocol

HeLa cells were plated in 6 wells plates or sterile microscopy slides (VWR, PA) and grown until confluence in DMEM/F12 medium (Invitrogen, NY) supplemented with 5% of fetal calf serum (FCS, Invitrogen, NY), 100 U/ml penicillin, 0.1 mg/mL streptomycin, and 0.29 mg/ml L-glutamine (Invitrogen, NY). Confluent cells were washed once with fresh media and then transduced with 2 mL of 1–1.5 TU/mL of EIAV-CMV-Null or UshStat vectors in complete media. After 2 hours, 2 mL of media was added and cells were grown for 48 hours, before collection for the experiments.

### Sample preparation and Western blot analysis

HeLa cells, 4 week old wild type neuroretina or RPE were homogenized in RIPA buffer (150 mM NaCl, 50 mM Tris, 1 mM EDTA, 1% NP-40, 0.5%, sodium deoxycholate, 0.1% SDS, pH 7.4) containing protease inhibitors, cleared by centrifugation, and 30–50 μg of protein used for Western blot analysis. For immunoprecipitation studies, 40 μl of protein-A sepharose beads (50% slurry, Sigma, MI) were incubated with 4 μg of rabbit anti-myosin VIIa (Novus Biologicals, CO) overnight at 4°C. The next day beads were washed once with RIPA and 400 μg of the HeLa lysate incubated overnight at 4°C with rocking. Beads were washed five times and then resuspended in sample buffer for Western blot analysis. Proteins were transferred to PVDF membranes and incubated for 2 hours at room temperature with 10% powdered milk blocking solution. Primary antibody dilution was 1∶500 in 5% milk blocking solution. After an overnight incubation at 4°C, membranes were incubated with the secondary antibody goat anti-mouse HRP-conjugated (Sigma, MO), dilution 1∶5,000 in 5% milk blocking solution for 1 hour at room temperature. Membranes were developed using ECL Plus (Pierce, IL). Membranes were stripped and re-probed with β-actin (Sigma, MO) as a loading control in the case of tissue lysates.

### Assessment for α-transducin translocation

Animals were kept overnight in cages in a lightproof darkroom. Following dark adaptation, the animals were placed in polystyrene immobilization tubes (Kent Scientific, Torrington, Connecticut) and exposed to 200 lux illumination for 10 minutes. These conditions were defined by our earlier studies [21]. Eyes were excised by cutting the optic nerve, a small incision was made between the cornea and the sclera and then they were immediately transferred to a 1.5 mL eppendorf tube containing 4% paraformaldehyde in 0.1 M sodium phosphate buffer pH 7.4 which was wrapped in aluminum foil. After at least 4 hours at 4°C, eyes were immersed in 30% sucrose, embedded in OCT medium and frozen at −80°C. Seven μm retinal sections were cut and stained with the specific antibodies.

### Immunohistochemistry

#### Immunostaining for α-transducin and GFP

Eye sections were rehydrated for 10 minutes at room temperature in phosphate-buffered saline (PBS) with 0.3% Triton X-100 (PBST) and incubated with the primary antibodies (mouse anti-α-transducin (clone TF15, Cytosignal, CA) at 1∶2,000 dilution and rabbit anti-GFP (Invitrogen, NY) at 1∶400 dilution), in PBST overnight at 4°C in a humidified chamber. After 3 washes in PBS at room temperature for 10 minutes, sections were incubated with the corresponding Alexa-Fluor conjugated secondary antibodies (Donkey anti-mouse Alexa Fluor 555 (Invitrogen, NY) at 1∶2,000 dilution and donkey anti-rabbit Alexa Fluor 488 (Invitrogen, NY) at 1∶300 dilution) in PBST for 1 hour at room temperature in a humidified chamber. After 3 more washes in PBS at room temperature for 10 minutes, slides were cover-slipped using Vectashield mounting medium (Vector Laboratories, CA).

#### Immunostaining for vector-derived myosin VIIa

Mouse tissue: The *shaker1* mice (*Myo7a^sh1-11J^*) used in these studies harbors an uncharacterized mutation in myosin VIIa such that a dysfunctional myosin VIIa is expressed. Therefore, to detect the expression of UshStat–derived human myosin VIIa, the monoclonal antibody against the human myosin VIIa (generated by our laboratories and described previously [27]) was diluted until the endogenous mouse myosin VIIa was no longer detected (1∶2000 dilution of affinity purified antibody stock solution at 1 mg/ml). Secondary antibodies were donkey anti-mouse Alexa Fluor 555 (Invitrogen, NY). Macaque tissue: Paraffin sections were heated at 65°C for 2 hours. De-waxed sections were rehydrated and rinsed in tap water. After re-hydration the sections underwent antigen retrieval for 3 minutes in a pressure cooker in Tris-EDTA buffer (10 mM Tris Base, 1 mM EDTA, pH 9.0), rinsed well in tap water and placed in blocking solution overnight (10% normal goat serum (S1000, Vector Laboratories, CA)). Mouse anti-myosin VIIa antibody was added at a dilution 1∶1000 in normal goat serum blocking solution and incubated for 2 hours. After 3 washes of 5 min each in PBS, slides were incubated with the secondary antibody Alexa-Fluor 488 1∶100 dilution (Invitrogen, NY). After several washes slides were mounted in Vectashield containing DAPI mounting medium (Vector Laboratories, CA). *HeLa cells*: HeLa cells grown on slides were fixed in cold-acetone for 10 minutes, air dried for 2 hours and re-hydrated with PBS. After permeabilization with 0.3% Triton X-100 in PBS and several washes, cells were blocked for at least 1 hour at room temperature in fish gelatin blocking solution (2% FCS, 0.3% fish gelatin in PBS). Primary antibody dilution was 1∶100 in fish gelatin solution overnight at 4°C. After 3 washes with PBS, slides were incubated with the secondary donkey anti-mouse Alexa Fluor 568, dilution 1∶500 in fish gelatin solution for 1 hour at room temperature. Slides were cover-slipped using Vectashield mounting medium with DAPI (Vector Laboratories, CA) and analyzed by confocal microscopy.

Confocal images were captured using a Zeiss AxioPlan 2IF MOT microscope interfaced with a LSM510 META confocal imaging system. Final figures were assembled using Adobe Photoshop and Illustrator software (Adobe Systems, CA).

### Assessment of light induced retinal degeneration

Following subretinal injection, mice were allowed to recover until they were 4–6 weeks old before light dependent retinal degeneration was assessed using one of two different approaches. They were either 1) light-adapted in continuous light (2000 lux) for 6 days, or 2) reared in a room for 3 months with 1500 lux 12 hour light/12 hour dark cycle (at cage level). Following light exposure, the animals were anesthetized with a ketamine-xylazine solution (i.p. 10 mg/ml and 1.6 mg/ml, respectively) followed by heart perfusion with 10 ml of PBS and then 10 ml of the fixative solution (4% paraformaldehyde in 0.1 M sodium phosphate buffer pH 7.4). Eyes were processed as described above and serial sections were immunostained for myosin VIIa and GFP or haematoxylin-eosin (H&E) stained. Confocal images were captured as described above. Only regions of the retina that were GFP-positive (indicative of human myosin VIIa expression) and therefore known to be transduced were used for data generation. The row of photoreceptor nuclei were counted in the serial sections stained with H&E and analyzed by light microscopy at a magnification of 40x. Measurements were taken within 0.2 mm from the place of injection. On a different set of experiments, non-injected wild type or *shaker1* mice were exposed to 15,000 lux illumination for 2 hours following dilatation of the pupils with 1% Tropicamide. For these and the TSSM-injected control retinas, ONL counts were taken from the same region as for the EIAV injected retinas.

The bar graphs represent means and standard deviations of at least four independent animals per group.

### Macaque toxicology and biodistribution study

Rhesus macaques were two to four years of age at the time of dosing. The right eye of each Rhesus macaque in the safety study was treated with either UshStat at a dose of 9.1×10^5^ transducing units per eye (TU/eye) or control buffer (TSSM) in a total volume of 100 μL, whilst the left eye remained untreated (**Table 1**).

**Table 1:** Study design for 3-month toxicology studies in Rhesus Macaques.

Group	Dose level TU/eye (right)	Article	Number of animals/group M/F	Sacrifice time point 3 months (D90) M/F
1	-	Formulation buffer	3/3	3/3
2	9.1×10^5^	UshStat vector	3/3	3/3

Abbreviations: TU, transducing unit; M/F, male/female.

For the GLP studies in the macaques the dosing apparatus consisted of a 41-gauge (G) needle connected to a micro-extension tube and a 1 mL luer lock syringe. The apparatus was prefilled with PBS and a small air bubble was created in the needle hub prior to filling with 100 μL of test or control article to minimize loss of vector in the dead volume of the apparatus. A light pipe was inserted transconjuctivally near the limbus using a 25G cannulation system. For the subretinal injection, the conjunctiva was opened and an insertion site opened using a V-lance to allow insertion of the wider barrel. Following injection, the 25G port was removed and the opening for the dosing needle sutured. The targeted dosing region was inferolateral or inferomedial to the optic nerve and was peripheral to the macula.

Samples of buffy coat, plasma, saliva and tear swabs/eye exudates were collected from each animal on days 3, 8 and 15 for qPCR or qRT-PCR analysis of vector presence. At the end of the study (92 days) a full macro- and microscopic examination was performed on a wide variety of tissues. Additional tissue and fluid samples were also collected for vector presence outside the ocular target compartment and periodic blood sampling was performed throughout the study to examine for possible antibody responses against either UshStat vector components or towards the transgenes. A full clinical chemistry and urine analysis was also performed on samples obtained 3 months after UshStat administration. In a separate study, qPCR was performed on retina and choroid samples from male Rhesus macaques treated with UshStat at a dose of 9.1×10^5^ transducing units in their right eye to confirm that the vector transduces cells within the retina following subretinal injection.

The assessment of toxicity against UshStat was based on mortality, clinical signs, body weight, and qualitative food consumption. In addition, regular in-life assessments of UshStat toleration were performed by slit-lamp biomicroscopy, indirect ophthalmoscopy, Tono-Pen Vet applanation tonometry for intraocular pressure (IOP) measurements, full-field ERG and color fundus photography. Anterior chamber and vitreous cell scores were assigned by using an estimate of cells per field of the focused slit lamp beam; 0 = no cells, 1+ = 5-25 cells (very slight), 2+ = 25–50 cells (slight), 3+ = 50–100 cells (moderate), 4+ = greater than 100 cells (severe). Aqueous flare refers to the ability to visualize the passage of a focused beam of light across the anterior chamber of the eye, this correlates with the protein content of the aqueous humor; 0 = no protein, 1+ = trace protein, 2+ = mild amount of protein apparent to experienced observer, 3+ = moderate protein opacity visible to the naked eye, 4+ = large amount of protein with a higher density than 3+.

### Statistical analysis

For light induced retinal degeneration studies, the results for at least 4 independent animals per group were statistically analyzed. Means and standard deviations for groups were compared by one way analysis of variance (ANOVA) to determine significant differences. A p value<0.05 was considered statistically significant.

## Results

To demonstrate the specificity of the myosin VIIa antibody developed at our institute [27], we performed western blot experiments and immunocytochemistry in mouse tissue and HeLa cells transduced with the EIAV-Null or UshStat (**Fig. 1A**). **Figure 1B** shows that two of the myosin VIIa variants previously described [28], can be detected by our antibody preparation in both RPE and neuroretina (NR). Immunoprecipitation of UshStat transduced cells lysates with a commercially available myosin VIIa antibody follow by immunoblot with the myosin VIIa antibody developed by our laboratory (IP/MYO7A) show a prominent band (red arrow) of similar molecular weight as the ones detected in mouse tissue. Immunocytochemistry experiments (**Fig. 1C–D**) show a positive signal only in those cells transduced with the UshStat construct (**Fig. 1D** **, red**). The results presented in **Figs. 1B–D**, demonstrate the specificity of our myosin VIIa monoclonal antibody.

### Subretinal delivery of UshStat rescues the shift in light thresholds for α-transducin translocation in *shaker1* mice

The transduction efficiency of EIAV-based vectors in mouse photoreceptors was determined by injecting P1-P3 mice with 10×1 μl of EIAV-CMV-GFP or EIAV-RK-GFP (9.4×10^6^ TU/mL and 6.0×10^8^ TU/mL, respectively). Transduction was analyzed 4 weeks after the injection in retinal cryosections. We observed at least 53% efficiency for EIAV-CMV-GFP (**Fig. S1A–B**) and a maximal efficiency of 44% for EIAV-RK-GFP (**Fig. S1C–D**). These transduction efficiency values were similar to the ones published by other groups [29], [30] using GFP-AAV (adeno-associated viral)-based vectors and demonstrated we were able to achieve similar levels of efficiency using a delivery vector with a higher cargo capacity. For subretinal co-transduction with myosin VIIa or Null vector and GFP, vectors were diluted accordingly (see Materials and Methods).

**Figure 2:**
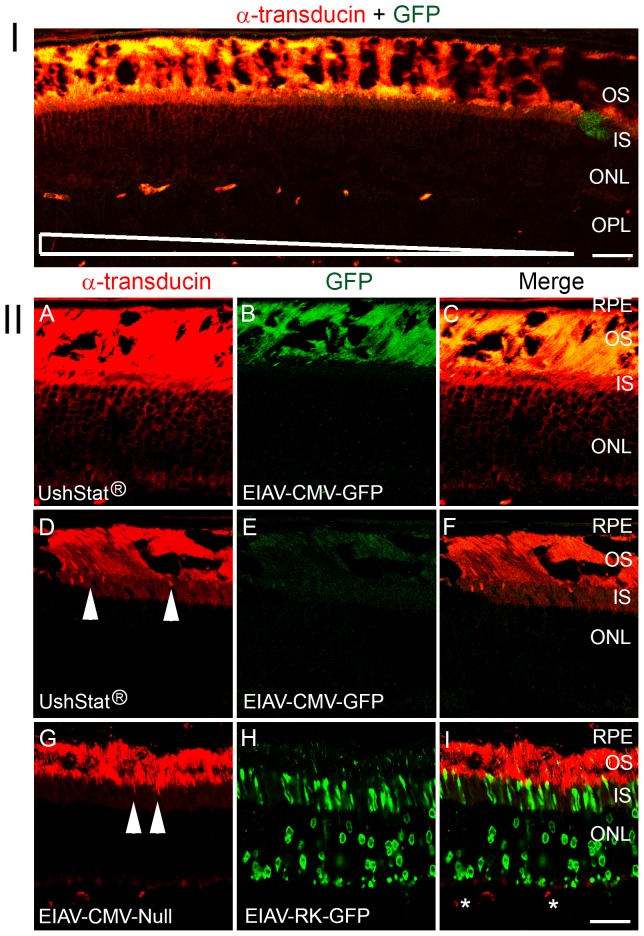
Expression of wild type myosin VIIa in *shaker1* retinas restores normal thresholds for light-induced α-transducin translocation. *Shaker1* mouse retinas were co-transduced by a subretinal injection of UshStat and EIAV-CMV-GFP or EIAV-CMV-Null and EIAV-RK-GFP (the GFP vector was used to identify the transduced region of the retina). After 4 weeks the animals were dark-adapted overnight, then light-adapted for 10 minutes under 200 lux illumination. Retinas were double immunostained with antibodies against GFP (green) and α-transducin (red). **Panel I**: Low magnification image of a retina transduce with UshStat and GFP, showing that the gradient in α-transducin translocation (left to right) parallels GFP expression (indicative of wild type myosin VIIa). Scale bar: 40 μm. **Panel II**: **A–C**: Representative examples of an EIAV transduced region of the retina (determine by the presence of GFP), presenting dual immunostaining for GFP and α-transducin (**C**). In the presence of wild type myosin VIIa, α-transducin is translocated to the inner segment (IS). **D–F**: An untransduced region of the same retina as in **A–C** does not show α-transducin translocation to the IS upon illumination. **G–I**: Retinas co-transduced with EIAV-Null-vector and EIAV-RK-GFP (photoreceptor cell-specific promoter) as a control for the effects of subretinal lentiviral transduction on α-transducin translocation. The region of the retina shown was transduced, evidenced by GFP expression in the photoreceptors (**H**) however there was no translocation of α-transducin to the IS (**G** and **I**). The qualitative results represented in each of the panels are representative images for at least three replicate experiments. RPE = Retinal Pigment Epithelium; OS = Outer Segments; IS = Inner Segments; ONL = Outer Nuclear Layer; OPL = Outer Plexiform Layer. Scale bar: 25 μm. Arrowheads in **D** and **G** indicate translocation of α-transducin in individual photoreceptors. Asterisks in I denote reactivity of the secondary anti-mouse antibody with circulation mouse IgGs present in the blood vessels.

Previously we demonstrated that the threshold of light required to activate the translocation of α-transducin from the outer to the inner segments of photoreceptors was elevated in *shaker1* mice [21]. To determine whether this phenotype could be rescued by gene therapy we subretinally treated mice with either the UshStat or EIAV-CMV-Null (control) vector and the EIAV-(CMV/RK)-GFP vector. Following light exposure at 200 lux for 10 minutes the eyes were harvested and the retinas assessed histologically for α-transducin translocation. Results in **Figure 2** demonstrate that we can rescue the phenotype associated with α-transducin translocation in the *shaker1* UshStat transduced retina. **Figure 2 panel I** shows a representative low magnification image of a retina co-transduced with UshStat and EIAV-CMV-GFP, showing that α-transducin translocation to the inner segment and outer nuclear layer parallels the gradient of GFP expression (yellow, indicative of wild type myosin VIIa expression). High magnification images (**Figure 2 panel II**), demonstrate that only the transduced (UshStat) regions of the retina determined by GFP immunostaining (**Fig. 2A–C**) showed a significant translocation of α-transducin from the outer to the inner segments of the photoreceptors relative to untransduced (control) regions of the same retinal section (**Fig. 2D–F**) or relative to retinas co-transduced with the EIAV-CMV-Null (control) vector and the EIAV-RK-GFP reporter gene (**Fig. 2G–I**). Controls show very little translocation of α-transducin from the outer segments to the inner segments (arrowheads). Note that because the vectors were co-injected with 10-fold more myosin VIIa expressing viral particles than GFP, the transduced-positive area will have more photoreceptors transduced with the wild type myosin VIIa than with GFP. The differences in the pattern of expression of GFP between **Fig.2B** and **Fig. 2H** are likely the result of different promoter usage.

These data demonstrate that an EIAV-based lentiviral vector expressing full length human myosin VIIa cDNA (UshStat) rescues the light threshold shift for α-transducin translocation in the *shaker1* mouse with this restoration to the normal phenotype only occurring in areas of the retina that were positively transduced with the wild type myosin VIIa (determined by the presence of GFP).

**Figure 3:**
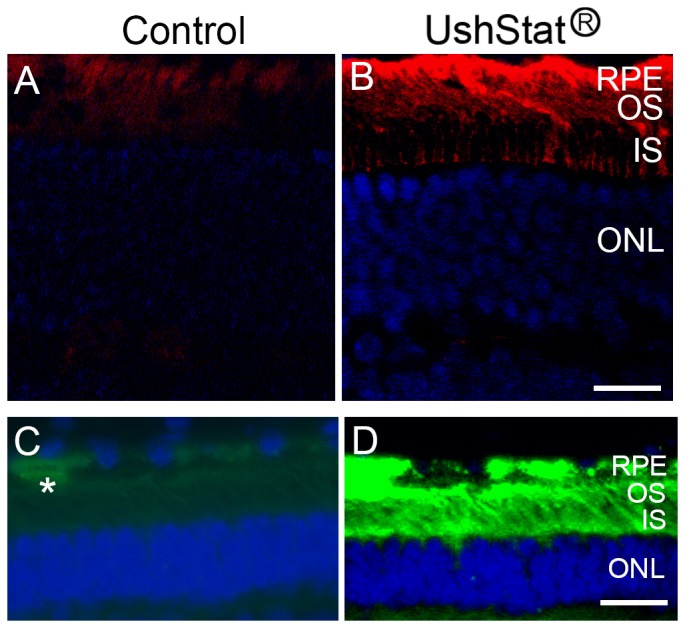
UshStat transduced mouse and monkey retinas showed expression of human myosin VIIa. **A–B**: Show myosin VIIa immunostaining of *shaker1* retinas transduced with the EIAV-CMV-Null vector (**A**) or UshStat (**B**). Myosin VIIa expression can be detected in the RPE, OS and IS regions. **C–D**: monkey retinas transduced with UshStat (**D**) showed high levels of human myosin VIIa in the RPE and moderate expression in outer and inner segments of the photoreceptor cells. Asterisk in **C** denotes weak immunostaining of the endogenous myosin VIIa in the RPE layer. Note that the anti-myosin VIIa antibody was titrated so as to only detect the exogenous overexpressed virus-derived wild type myosin VIIa (**B, D**) and not the endogenous protein (**A, C**). Scale bars: 25 μm.

Mouse UshStat transduced retinal sections were immunostained with antibodies for myosin VIIa to verify expression of the human myosin VIIa (**Fig. 3A–B**). Macaque paraffin-embedded section from retinas transduced with UshStat vector also showed expression of the human myosin VIIa, demonstrating primate retinas can be efficiently transduced by lentiviral-based vectors (**Fig. 3C–D**). UshStat transduced retinas (**Fig. 3B, D**) showed strong myosin VIIa immunostaining in the RPE and modest immunostaining in the outer and inner segments of photoreceptor cells [31], [32]. The control transduced retinas (**Fig. 3A, C**), show little to no immunostaining for myosin VIIa under conditions that would only minimally detect the endogenous myosin VIIa.

In a previous report using the same monoclonal antibody, Soni et al. [27] observed expression of myosin VIIa in the RPE only, while we are also detecting myosin VIIa in the photoreceptors (**Fig. 3B, D**). This difference is likely due to overexpression of the human myosin VIIa driven by the CMV promoter.

#### Sub-retinal delivery of UshStat rescues the light induced photoreceptor degeneration phenotype in shaker1 mice

**Figure 4:**
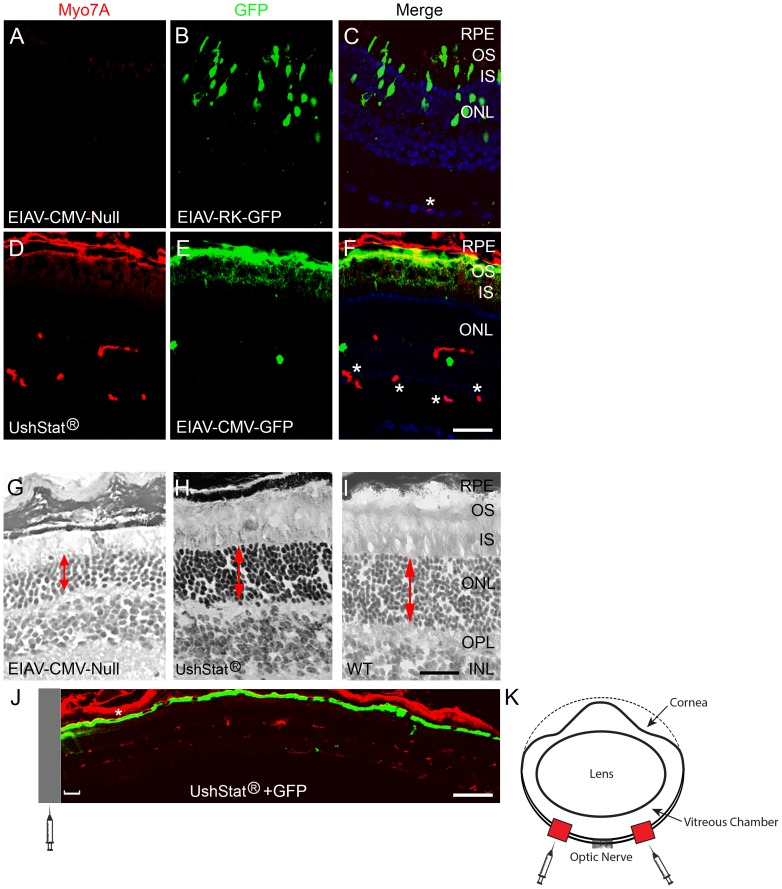
Subretinal injection of *shaker1* retinas with UshStat rescues the light-induced photoreceptor degeneration phenotype. Retinas were co-transduced with EIAV-CMV-Null vector and EIAV-RK-GFP (**A–C**) or UshStat and EIAV-CMV-GFP (**D–F, J**). After 4 weeks, mice were exposed to 6 days continuous light at 2000 lux illumination. Retinas were harvested and dual immunostained for myosin VIIa (red) and GFP (green) (**A–F, J**). Immunostaining conditions were chosen as to only detect the exogenous overexpressed myosin VIIa. **G–H**: Eosin and hematoxylin histochemical staining of *shaker1* mouse retinas transduced with either the EIAV-CMV-Null vector (**G**) or UshStat (**H**). **I**: Eosin and hematoxylin staining of wild type (untransduced) retina for comparison of relative light dependent degeneration of the ONL that is typically observed (double headed arrow). **J**: Low magnification image of an UshStat+GFP co-transduced retina, showing the area used for the studies (bracket) relative to the area of injection (syringe). This area corresponds to the juxtaposed ∼0.2 mm from the site of injection. **K**: Schematic representation of the area of injection represented by red boxes, where right box area corresponds to the injection in the right eye and left box area injection in the left eye. The inferior retina was always used for the injections and ONL counting. Scale bars **A–F**: 25 μm, **G–I**: 50 μm. **J**: 90 μm. Labels are as in Figure 2. Asterisks in **C**, **F** and J denote reactivity of the secondary anti-mouse antibody with circulation mouse IgGs present in the blood vessels.

In earlier work we demonstrated that *shaker1* mice were susceptible to photoreceptor degeneration from either an acute (2000 lux continuous for 6 days) or a chronic (1500 lux light/dark cycle for 3–6 months) light insult, whilst under these same conditions, strain and age matched wild type mice showed no meaningful loss of photoreceptors [21]. Here, we investigated the ability of the UshStat vector to rescue this phenotype following an acute light insult of continuous exposure to 2000 lux for 6 days. *Shaker1* mice were subretinally injected with either the UshStat, EIAV-CMV-Null, or control buffer (TSSM) and at the end of the study the eyes were harvested for assessment of light induced retinal degeneration. The results in **Figure 4** show typical retinas isolated from *shaker1* mice that had been co-transduced with the EIAV-CMV-Null vector and EIAV-RK-GFP (**Fig. 4A–C**) or UshStat vector and EIAV-CMV-GFP (**Fig. 4D–F**) following 6 days continuous light exposure (2000 lux). At the end of the 6 days continuous light exposure at 2000 lux the UshStat treated eyes show a marked increase in the ONL thickness (**Fig. 4H** and **Fig. S2**) compared to *shaker1* mouse eyes that had been treated with the EIAV-CMV-Null vector (**Fig. 4G** and **Fig. S2**), where the double-headed arrows denote the relative thickness of the outer nuclear layers. Wild type retina was included for comparison with the myosin VIIa transduced *shaker1* retinas (**Fig. 4I**). **Figure 4J** shows a low magnification image of an UshStat transduced retina immunostained for myosin VIIa and GFP, depicting the region used for these experiments (bracket) relative to the site of sub-retinal injection (syringe in **Fig.4J and 4K**). This distance was always within 0.2 mm from the site of injection and away from the optic nerve.

**Figure 5:**
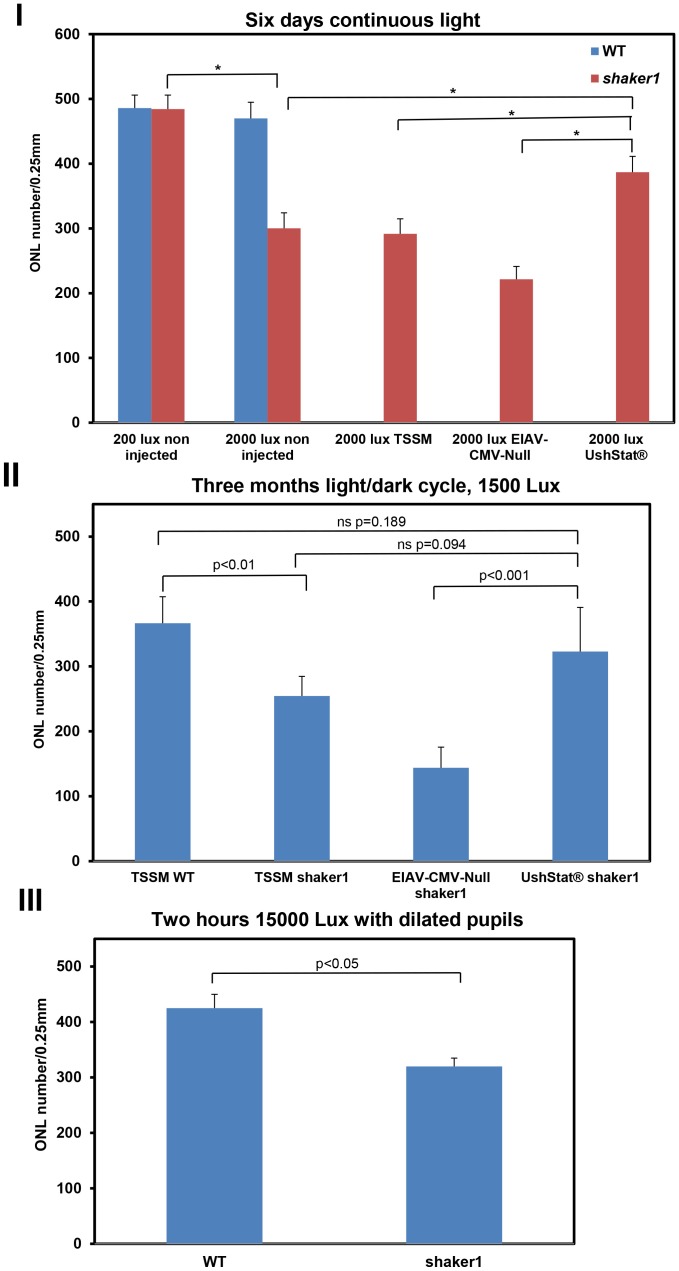
Quantitative analysis of UshStat-mediated rescue of the light induced photoreceptor degeneration phenotype in *shaker1* mice. **Graph I**. *Shaker1* mice were transduced with either UshStat or EIAV-CMV-Null vector (in combination with the EIAV-CMV/RK-GFP as previously described to identify regions of the retinas transduced). Following 4 to 6 weeks recovery, the animals were subjected to 6 days continuous light exposure to 2000 lux and photoreceptor cell numbers were counted as described in the methods section. **Graph II**. Wild type or *shaker1* mice were transduced with TSSM formulation buffer or either UshStat or EIAV-CMV-Null vector in combination with EIAV-CMV/RK-GFP. Following 4 to 6 weeks recovery, the animals were subjected to 3 months 12 h/12 h light dark cycle with 1500 lux illumination and the photoreceptor cell numbers counted. At least 5 retinas per group were examined. **Graph III**. Either *shaker1* mice or strain/age matched (8 to 10 week old) wild type mice were subjected to 15000 lux illumination for 2 hours following dilation of the pupils with 1% Tropicamide. There were at least 4 (for most cases greater than 5) replicates per group. Statistical analysis between groups was determined by analyzing the mean and standard deviations for each group using one way analysis of variance (ANOVA). Asterisks denote statistically significant differences between groups (p<0.001).

The ONL thickness was quantified from transduced regions of the retina in at least four individually treated mice (**Figure 5** **graph I, Fig. S2**). Under the normal *vivarium* 200 lux light/dark cycle conditions, there is no significant decrease in the ONL numbers comparing wild type and s*haker1* groups (490.3±19.5 *versus* 484±21.6). Consistent with our earlier studies, 2000 lux illumination for six continuous days did not significantly affect photoreceptor cell numbers in wild type mice, but significantly reduced the number of photoreceptors in *shaker1* mice (475.6±29.5 *versus* 300.2±23.9). Transduction of *shaker1* mice with the EIAV-CMV-Null control vector lead to similar levels of light dependent ONL degeneration as compared to untransduced *shaker1* mice. However transduction with the UshStat vector resulted in a significant increase in photoreceptor cell numbers (386.8±24.6) compared to either the TSSM-injected (291.75±23.1) or the EIAV-CMV-Null (221.6±19.6) vector-treated *shaker1* groups, demonstrating that under these conditions, UshStat is capable of significant rescue the light-induced photoreceptor degeneration phenotype in *shaker1* mice.

Under a second set of conditions we investigated the ability of the UshStat vector to rescue the light-dependent photoreceptor degeneration phenotype following a chronic light insult of a 12 h/12 h light/dark cycle under 1500 lux illumination for 3 months. (**Figure 5** **graph II**). Unsurprisingly the subretinal administration procedure *per se* causes damage to the outer nuclear layer as seen in the TSSM treated *shaker1* mouse (**Fig. S2**) and this is further exacerbated by the administration of the control EIAV-Null vector in *shaker1* mouse (**Figure 5** **graph II** and **Fig. S2**). This damage is most probably due to the young age (P1-3) (neonatal) of the animals being treated and the small size of the eye relative to the needle and means that the true comparison should be made between the UshStat and EIAV-CMV-Null treated *shaker1* mice. The results illustrate that *shaker1* group treated with control (TSSM) buffer showed significantly more light induced retinal degeneration (254.5±30.3) when compared to the control (TSSM) treated wild type group (366.5±40.9). Importantly, subretinal injection of *shaker1* mice with the UshStat vector (**Fig. S2**) showed significant protection of photoreceptors, resulting in twice the number of photoreceptors (323.0±67.8) compared to *shaker1* mice treated with the EIAV-CMV-Null vector (144.0±31.8). When the UshStat *shaker1* group was compared to the TSSM *shaker1* group, the results were not significant (but trended towards significance), likely owing to the negative effects of the EIAV vector on photoreceptor cell health in these neonatal mice as exemplified by the TSSM *shaker1* group to the EIAV-CMV-Null *shaker1* group.

These findings regarding moderate light induced retinal degeneration in s*haker1* mice are in contrast to an earlier report which demonstrated that a *shaker1* mouse model was resistant to light-induced retinal degeneration under conditions of pupil dilation followed by exposure to 15,000 lux illumination for 2 hours [33]. While this extreme light exposure is different from the ones used in our study, the results are in stark contrast to the light sensitivity observed in our *shaker1* mouse model. To address this inconsistency we repeated these experiments with our *shaker1* mouse model using the exact conditions described in the earlier report [33]. The results in **Figure 5** **graph III** demonstrate that our *shaker1* mice show significantly more retinal degeneration following exposure to 15,000 lux for 2 hours compared to strain/age-matched wild-type mice (425±25 *versus* 312±13). The mice used in our study and those used in the earlier report both harbor the L450 genotype for RPE65. These data confirm the light-induced photoreceptor degeneration phenotype in the *shaker1* mice (*Myo7a^sh1-11J^*) used in this study.

Collectively, these data demonstrate that under a chronic light/dark cycle UshStat is able to rescue the light dependent retinal photoreceptor degeneration phenotype in *shaker1* mice.

### Macaque GLP toxicology study to evaluate UshStat

The macaque GLP study was designed to examine ocular toleration to subretinally-dosed UshStat vector at a dose of 9.1×10^5^ TU/eye. This dose of UshStat vector was determined based on a previous exploratory study in Rhesus macaques and it is the highest ocular tolerated dose that was evaluated. Immunohistochemistry analysis of UshStat injected macaque retinas (**Fig. 3D**) showed high expression of myosin VIIa in the RPE and moderate expression in the outer and inner segments of photoreceptor cells compare with TSSM injected eyes (**Fig. 3C**).

#### Clinical Assessments

There were no unscheduled deaths that were related to subretinal administration of UshStat or control buffer nor were there any statistically significant differences in body weights and relative organ weights at the scheduled necropsy at 3 months in macaques treated with UshStat as compared to control animals dosed subretinally with buffer. No treatment-related changes in blood chemistry were observed in the UshStat-treated animals at any of the sampling time points, nor was there any evidence for changes in red cell or white cell counts. UshStat did not change blood clotting times and there were no significant treatment-related microscopic observations in non-ocular tissues in the macaques.

#### Clinical Ophthalmic Observations

**Figure 6:**
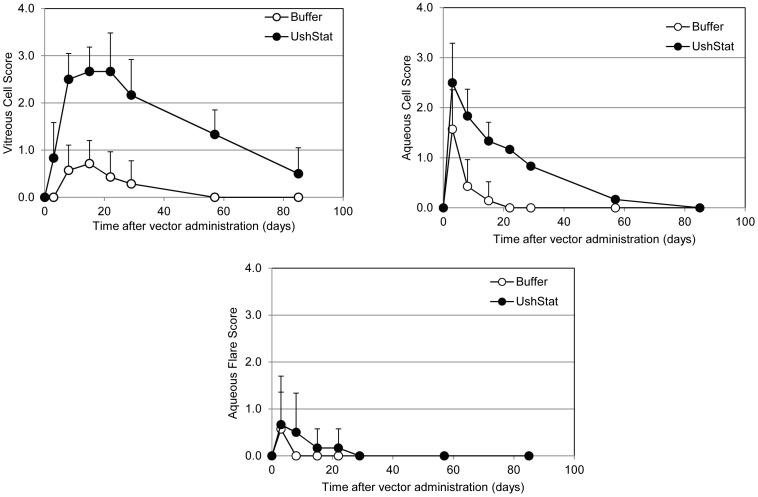
In vivo ocular inflammatory scores in Rhesus macaques following sub-retinal delivery of either UshStat or TSSM buffer. Inflammatory changes were assessed by slit-lamp biomicroscopy and indirect ophthalmoscopy. The clinical changes were graded at four levels of severity; +1 = very slight, +2 = slight, +3 = moderate, +4 = severe.

Subretinal injections resulted in well-developed subretinal blebs in all but one NHP treated right eye with the bleb no longer present by the end of the study. Slit-lamp biomicroscopy showed that UshStat treated eyes displayed an increased severity and duration of vitreal opacity compared to control buffer treated eyes (**Figure 6**) that resolved in all but one animal by the end of the study. In this animal the subretinal bleb was not properly created and subsequently some of the vector dose escaped into the vitreous, however the severe vitreal opacity in this animal at day 22 had significantly improved by the end of the study. Slight to moderate anterior cell scores were observed in UshStat-treated animals at day 3 that resolved by the end of the study. Control buffer treated eyes had slight vitreal/anterior cell scores that resolved by the end of the study. Very-slight to slight aqueous flare scores were seen in most of the animals with a higher incidence and duration in the UshStat-treated group, this resolved by day 29.

**Figure 7:**
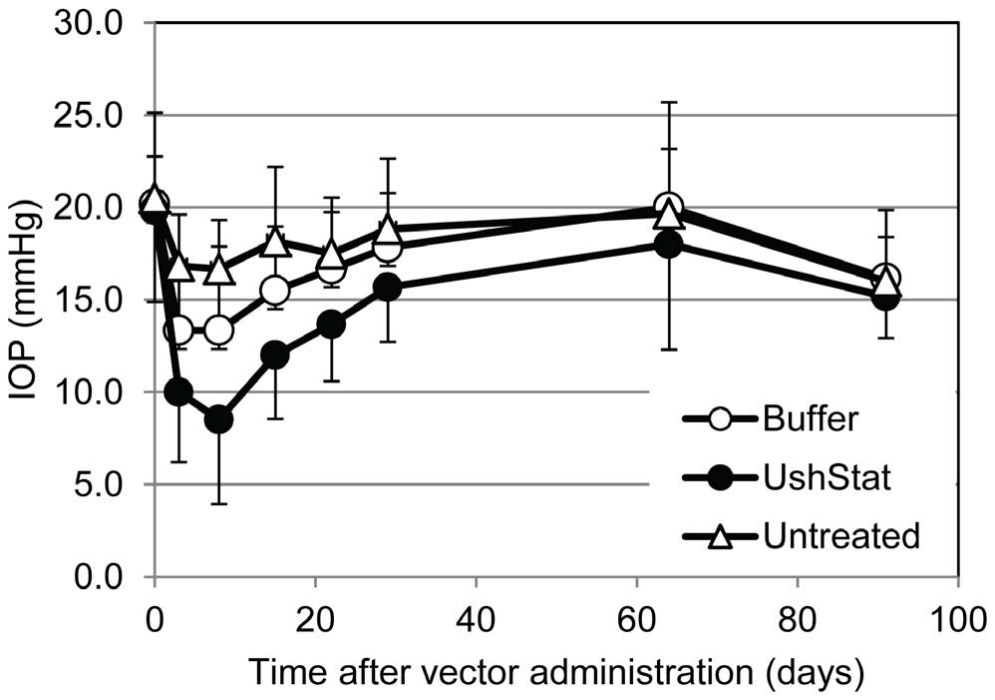
The mean intraocular pressure (IOP) measurements in Rhesus macaques following sub-retinal delivery of UshStat, TSSM buffer or untreated. Time points for each group are shown +/− standard deviation.

Transient reductions in the IOP readings were noted in both UshStat and control buffer-treated right, but not their corresponding untreated left eyes, which lasted for approximately 3–4 weeks after dosing and subsequently returned to the normal range (15–20 mmHg) for the remainder of the study (**Figure 7**). The magnitude of the fall in the IOP was slightly greater in the UshStat-treated right eyes, versus buffer dosed eyes and is most likely a reflection of the greater level of inflammatory response observed in the anterior segment of the UshStat-treated eyes.

No clinically significant changes in ERG responses were observed between the two groups but pigment changes in the retina/choroid associated with dose bleb formation and needle entry site were observed in treated eyes of both dose groups. Very slight to moderate irregular pigmentation of the macula/fovea associated with physical separation of the neurosensory layer from the retinal pigment epithelium during bleb formation were noted in two UshStat-treated eyes and five vehicle-treated eyes and remained present until the end of the study.

#### Ocular Histology

All microscopic findings including minimal to slight segmental hyperpigmentation/hypertrophy/hyperplasia of the RPE, retinal oedema, mononuclear infiltration and slight axon degeneration were observed in both the UshStat and control buffer treated macaque eyes. The findings were localized to the subretinal injection site and were considered an effect of the injection procedure and delivery of a subretinal volume.

#### Biodistribution, Persistence and Shedding of UshStat

By measuring the amount of UshStat RNA (UshStat vector particles contain two copies of the genome) or DNA (UshStat integrates into the target cell chromosome) an estimate of vector distribution can be made. Various fluids, tissues and buffy coat were analyzed by qPCR at selected time points from the macaque study to determine the biodistribution, persistence and shedding of the UshStat vector following subretinal delivery. Vector particle dissemination and/or persistence were assessed by analyzing RNA extracted from plasma samples for the presence of UshStat-derived EIAV Ψ sequences. The same qRT-PCR assay was used to assess vector shedding from RNA extracted from plasma, swabs of saliva and tears, and UshStat-treated right eye vitreous fluid. Biodistribution was assessed by analyzing DNA extracted from various tissues and buffy coat samples for the presence of UshStat-derived EIAV packaging signal (Ψ) sequences. Vector presence in target tissues was measured from samples from a separate macaque study.

In this study, in-life sampling indicated that no vector sequences were detected in RNA extracted from saliva swabs and right eye tear swabs at day 3 (**Table 2**). The RNA vector sequences that were detected in plasma samples were non-quantifiable in a minority of animals at day 3. By day 8, no RNA vector sequences were detected in any of the plasma samples.

**Table 2:** Summary of results from UshStat treated macaques Buffer treated macaques.

Method	Tissue/sample	Time point/number of UshStat-treated animals testing positive				
		D3	D8	D15	D29	D92
RNA analysis by qRT-PCR	Plasma	1/6^b^	0/6	-	-	-
	Saliva swab	0/4^c^	0/2^c^	-	-	-
	Right eye tear swab	0/6	-	-	-	-
DNA analysis by qPCR	Buffy coat	3/6^a^	2/6^d^	0/6	-	-
	Ovary	-	-	-	-	0/3
	Testes	-	-	-	-	0/3
	Liver	-	-	-	-	1/6^e^
	Spleen	-	-	-	-	1/6^f^
	Brain	-	-	-	-	0/6
	Optic chiasm	-	-	-	-	0/6
	Right eye optic nerve	-	-	-	-	0/6
	Right eye retina choroid	-	-	-	-	3/4
	Right eye sclera	-	-	-	-	2/4
		**Number of buffer-treated animals tested positive/number tested**				
RNA analysis by qRT-PCR	Plasma	0/6	-	-	-	-
DNA analysis by qPCR	Buffy coat	-	0/6	-	-	-
	Liver	-	-	-	-	0/6
	Spleen	-	-	-	-	0/6

Key: ^a^positive signal below the LLOQ for 3/6 animals (LLOQ 10 copies, copies detected ∼1 to ∼3). ^b^positive signal below the LLOQ for (Lower Limit Of Quantification) 1/6 animals (LLOQ 100 copies, copies detected ∼65). ^c^saliva swabs were only available from 4 animals at day 3 and 2 animals at day 8. ^d^positive signal below the LLOQ for 2/6 animals (LLOQ 10 copies, copies detected ∼2). ^e^positive signal below the LLOQ for 1/6 animals (∼1 copy detected). ^f^positive signal below the LLOQ for 1/6 animals (LLOQ 10 copies, copies detected ∼2).

DNA vector sequences were detected in half of the buffy coat samples collected from animals at day 3 and in a third of the buffy coat samples collected from animals at day 8. The level detected was always at a level that was non-quantifiable (below the Lower Limit Of Quantification (LLOQ less than 10 copies). By day 15, no DNA vector sequences were detected in any of the buffy coat samples.

No vector sequences were detected in the following tissue sample types at necropsy on day 92: brain, ovary, testis, optic chiasm, right optic nerve. Non-quantifiable vector sequences, at a level that was below the LLOQ, were only detected in the spleen and liver tissues collected from 1 out of the total of 6 animals. Vector DNA was not detected in spleen and liver from the remaining 5 animals.

In a separate study quantifiable vector sequences were found in three out of the four NHPs within the retina/choroid samples following subretinal delivery of UshStat at the end of the 3-month study (**Table 2**). The reason that one of the NHP eyes was negative for UshStat vector sequences is likely due to the rupture of the subretinal bleb upon dosing. Post-rupture, the vitreous appeared hazy due to the presence of the test article and this subsequently correlated with an unusually high level of ocular inflammation in this one animal and the detection of vector sequence in the vitreous at the end of the study.

#### Immunogenicity

**Figure 8:**
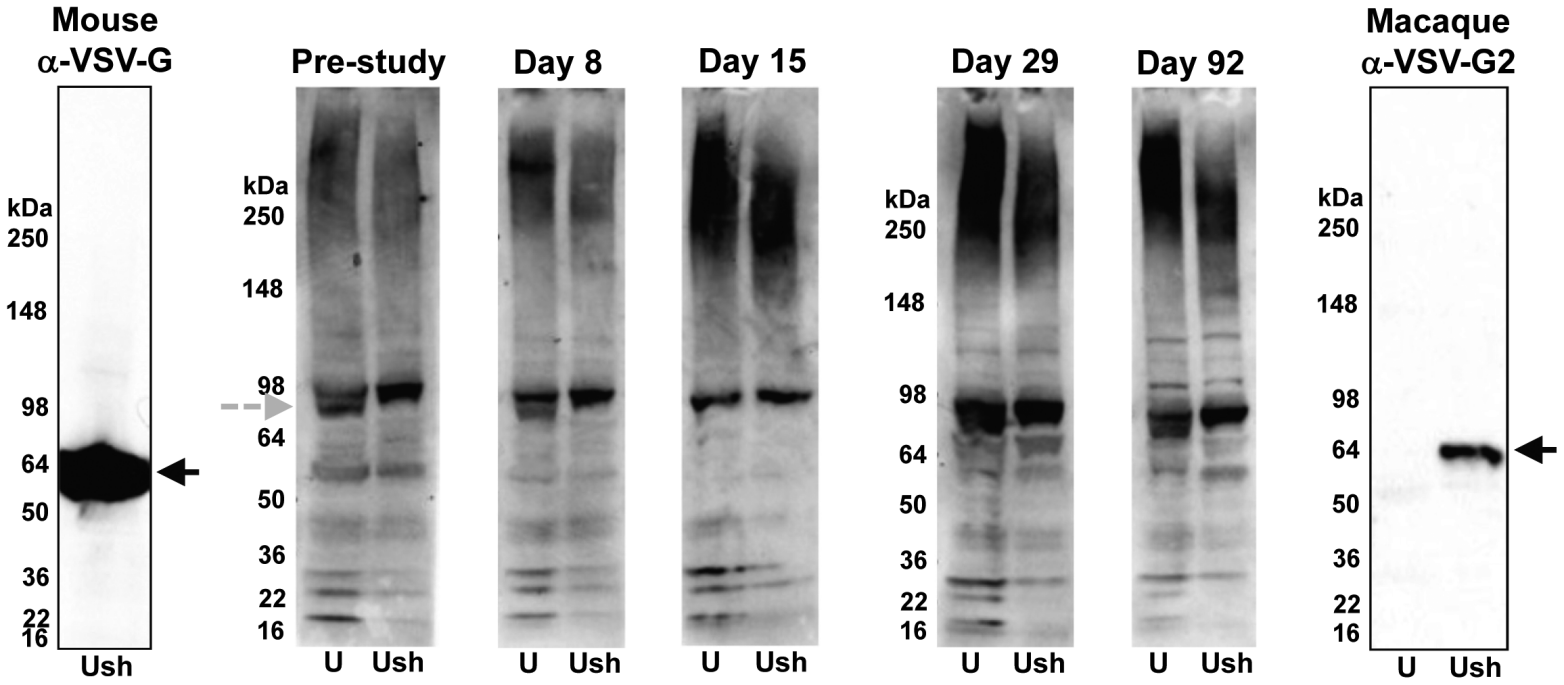
Representative Western blot of serum samples recovered from a macaque treated subretinally with UshStat. The serum samples were collected pre-treatment (pre-study) and 8, 15, 29 and 92 days after subretinal administration of the vector. The serum was tested against non-reduced lysates from untransfected HEK293T cells (U) and HEK293T cells transfected with the plasmids required to produce UshStat by Western blot analysis. Each serum samples was diluted 1/50. Antibody responses against a HEK293T-associated antigen could be detected in various pre and post study samples (grey arrow) in both UshStat and buffer treated animals and therefore may represent antibody responses stimulated by environmental factors rather than administration of UshStat. Molecular weights are indicated. Controls for the detection of VSVG (indicated by the black arrow) include a mouse monoclonal anti-VSVG antibody (left) and a macaque anti-VSVG positive control serum (right).

Immunological assessments of serum samples up to 3 months in the NHP GLP study did not show a humoral antibody response towards the MYO7A transgene, the VSV-G or any of the other UshStat vector components. An antibody response to the packaging cell HEK293T-associated proteins was detected in some of the serum samples (**Figure 8**). This was attributed to prior exposure of the animals to similar antigens before the study was initiated and was not considered a treatment-related phenomenon.

## Discussion

We recently described that USH1B mice have a robust light-dependent retinal phenotype [21]. Specifically, *shaker1* mice showed elevated light thresholds for induction of α-transducin translocation from the outer to the inner segments of rod photoreceptors, and sensitivity to moderate light-induced retinal degeneration. These findings imply that the USH1B retinal phenotype (at least in mice) is light-dependent. Importantly, these findings provide a model that can be used to evaluate the effects of novel therapies aimed at ameliorating the retinal phenotype in individuals with USH1B. In this study we demonstrate that UshStat, an EIAV-based lentiviral gene therapy vector harboring a full length human myosin VIIa expression cassette under the control of a constitutive CMV promoter, restores the α-transducin translocation threshold and reduces the light-dependent photoreceptor degeneration phenotypes in *shaker1* mice.

Neonatal *shaker1* mice were chosen for evaluation of the impact of the UshStat vector on retinal phenotypes since efficient EIAV-mediated gene transfer to photoreceptors has previously been shown in post-natal day 4 mice such that it rescued the phenotype in the *Abca4-/-* knockout mouse model of Stargardt disease [34]. The efficiency of photoreceptor gene transfer in adult mice with EIAV and other lentiviral based vectors appears to be lower [35]–[37], however, this does not reflect the transducing capabilities of the EIAV vector in other animals since photoreceptor transduction has been shown in Rhesus macaque, and the rabbit following subretinal delivery of an EIAV vector [38].

The first demonstration of a functional rescue by way of gene therapy in *shaker1* mice, utilized an HIV-based lentiviral vector expressing human *MYO7A* (HIV-MYO7A) that rescued two other *shaker1* phenotypes when introduced via a subretinal injection [28], [39]. The HIV-MYO7A vector corrected the abnormal phagocytosis and melanosome motility that caused an apical localization of the melanosomes in treated *shaker1* mice [40]. More recently, the FERM domain of myosin VIIa was shown to play a critical role in melanosome transport [41]. In this same study it was shown that the accumulation of opsin in the transition zone of the photoreceptor cilia of *shaker1* mice, an observation attributed to a functional role for myosin VIIa as a selective barrier to transport of membrane proteins to the outer segments of photoreceptors, was also corrected by the HIV-MYO7A vector [41]. Recently, two different groups demonstrated it is possible to effectively deliver large genes, like MYO7A, using AAV-based vectors, by single or dual AAV complementation. These works showed a correction of b-wave responses and in the melanosome and opsin distribution in unchallenged retina upon myosin VIIa transduction [29]–[30], [42]. Altogether, these observations suggested that gene therapy might also ameliorate the photoreceptor cell phenotypes, consistent with our results. Importantly, in the present study, by challenging the retina with moderate light, we demonstrate that UshStat vector rescues the light induced retinal degeneration phenotype in *shaker1* mice, which suggests that this therapy might protect against light dependent photoreceptor degeneration associated with *retinitis pigmentosa* in USH1B individuals.

In addition to melanosome trafficking and phagocytosis, it was recently shown that myosin VIIa also functions in the light-dependent translocation of RPE65 to the central region of the RPE [33]. This translocation appears to be important in optimizing phototransduction, and is also observed in human USH1B, and thus may reflect a functional property that could contribute to the development of *retinitis pigmentosa* in Usher patients [43]. While myosin VIIa may be directly involved in these RPE cell functions, its role in α-transducin translocation in photoreceptor cells is, more likely, indirect through a still unknown upstream mechanism. Thus our work here and this earlier work underscore the fact that myosin VIIa has cellular functions in both the RPE and photoreceptor cell layers of the retina. This begs the question as to whether rescue of the light induced retinal degeneration phenotype requires expression in one or both cellular compartments. Such studies may be possible using vectors that restrict MYO7A expression to either RPE or photoreceptor cells [30] and this would be useful for elucidating whether functional complementation via myosin VIIa expression directed specifically to either cell compartment is sufficient to rescue α-transducin translocation thresholds and light dependent retinal degeneration in *shaker1* mice. Such information would help begin to explain the biological function of myosin VIIa critical for photoreceptor cell health.

The GLP UshStat toxicology, biodistribution, shedding, and immunogenicity study in Rhesus macaques showed that a single subretinal injection of UshStat was safe and well-tolerated with slight-moderate transient ocular inflammation. The eye is anatomically separated from the rest of the body via the blood retina barrier and it is an immune-privileged compartment making it an attractive target for gene therapy applications. This is consistent with the UshStat vector remaining confined to the ocular space and a lack of a humoral antibody response to either the *MYO7A* transgene or vector components. These observations are similar to those observed with related ocular EIAV-based gene therapies, RetinoStat^®^ and StarGen™ which are both delivered subretinally and are currently employed in clinical trials to treat patients with either the ‘wet’ form of age-related macular degeneration or Stargardt disease respectively [26], [34], [38]. While the UshStat-treated eyes displayed an increased severity and duration of vitreal opacity, these responses resolved prior to the end of the study and did not lead to any histopathological events.

In summary, we have shown that subretinal delivery of UshStat is able to restore the α-transducin translocation phenotype in the *shaker1* mouse model and protect the photoreceptors from moderate light intensity damage. Assessment of safety in a 3 month GLP primate study showed this vector is safe and well-tolerated, elicits minimal immune responses, and is confined to the ocular compartment following subretinal administration. These data provided support for the initiation of the first-in-man clinical trial of UshStat in patients with Usher syndrome 1B (ClinicalTrials.gov identifier: NCT01505062) that is currently underway.

## Supporting Information

Figure S1 **Transduce efficiency studies.** S*haker1* retinas were transduced with 10-fold EIAV-CMV-GFP (**A–B**, 9.4×10^6^ TU/mL) or EIAV-RK-GFP (**C–D**, 6.0×10^8^ TU/mL). **A–B**: Low (**A**) and high (**B**) magnification images showing GFP presence in RPE, OS, IS, ONL and OPL. **C–D**: Low (**C**) and high (**D**) magnification images showing GFP presence only in the photoreceptor cell layer. Scale bars: **A** = 90 μm. **B, D** = 15 μm. **C** = 50 μm.
(JPG)

Figure S2 **UshStat vector rescues the light induced photoreceptor degeneration phenotype under different light conditions.** Representative images showing light induced photoreceptor degeneration results after 6 days continuous 2,000 lux (**B, C**) or 12 hr/12 hr dark/light cycle for 3 months. **A**: *shaker1* non-injected retina 200 lux light adaptation. **B,C**: *shaker1* retinas injected with the Null (**B**) or the UshStat vectors (**C**). **D**: shaker1 retinas injected with the formulation buffer TSSM. E, F: *shaker1* retinas injected with the Null (**E**) or the UshStat vectors (**F**). Scale bar: 35 μm.
(JPG)
