# Supplementary figures and images for: EIAV-Based Retinal Gene Therapy in the shaker1 Mouse Model for Usher Syndrome Type 1B: Development of UshStat
Source: PLoS One. 2014 Apr 4;9(4):e94272. doi: 10.1371/journal.pone.0094272 (PMC3976400; doi:10.1371/journal.pone.0094272)

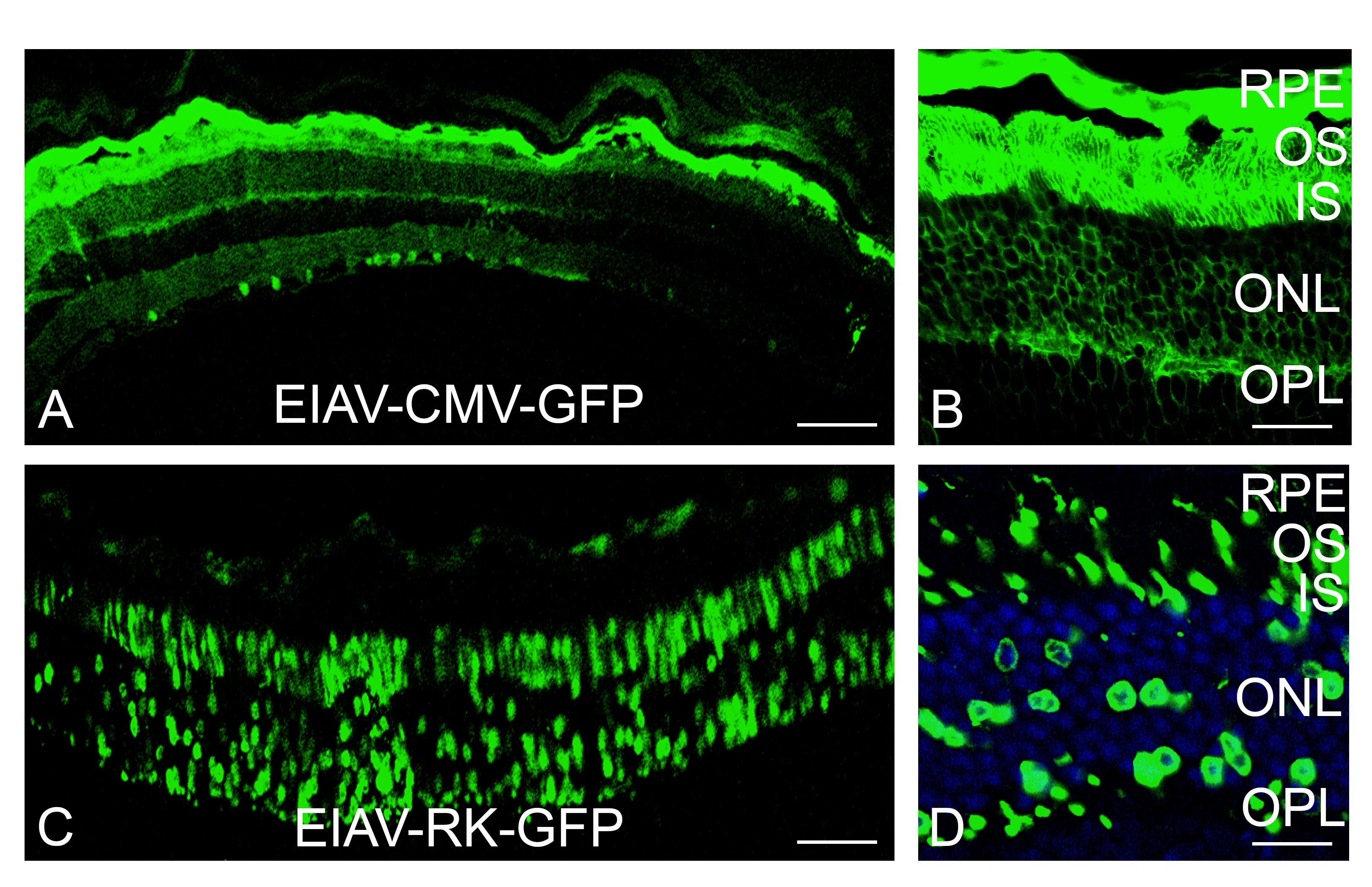

Supplement: Figure S1 — Transduce efficiency studies. Shaker1 retinas were transduced with 10-fold EIAV-CMV-GFP (A–B, 9.4×10^6^ TU/mL) or EIAV-RK-GFP (C–D, 6.0×10^8^ TU/mL). A–B: Low (A) and high (B) magnification images showing GFP presence in RPE, OS, IS, ONL and OPL. C–D: Low (C) and high (D) magnification images showing GFP presence only in the photoreceptor cell layer. Scale bars: A = 90 μm. B, D = 15 μm. C = 50 μm. (JPG) [file pone.0094272.s001.jpg]

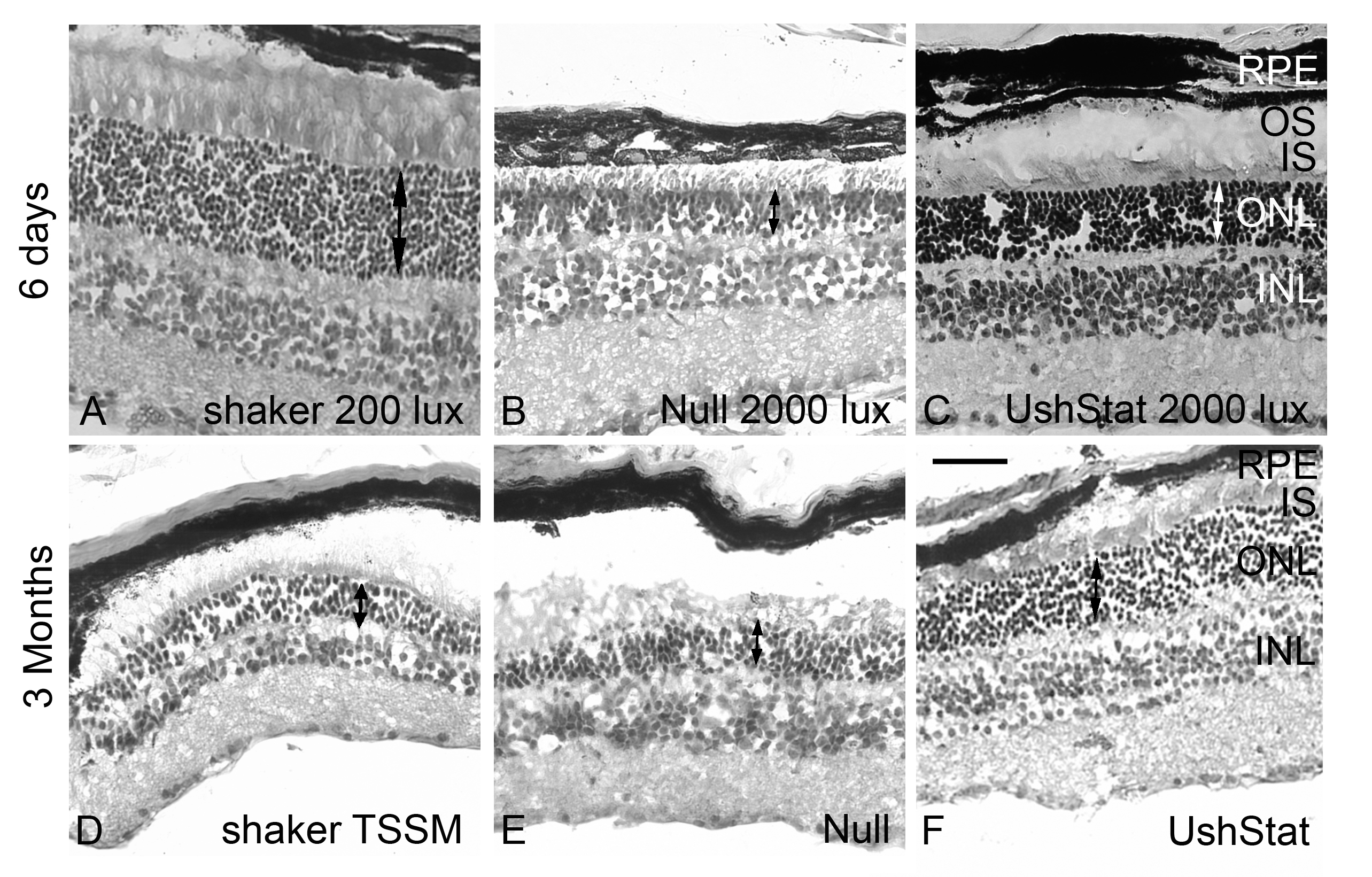

Supplement: Figure S2 — UshStat vector rescues the light induced photoreceptor degeneration phenotype under different light conditions. Representative images showing light induced photoreceptor degeneration results after 6 days continuous 2,000 lux (B, C) or 12 hr/12 hr dark/light cycle for 3 months. A: shaker1 non-injected retina 200 lux light adaptation. B,C: shaker1 retinas injected with the Null (B) or the UshStat vectors (C). D: shaker1 retinas injected with the formulation buffer TSSM. E, F: shaker1 retinas injected with the Null (E) or the UshStat vectors (F). Scale bar: 35 μm. (JPG) [file pone.0094272.s002.jpg]
